# A Comparative Analysis of the Venom System between Two Morphotypes of the Sea Anemone *Actinia equina*

**DOI:** 10.3390/ani14060981

**Published:** 2024-03-21

**Authors:** Maria Alcaide, Inês Moutinho Cabral, Lara Carvalho, Vera M. Mendes, António P. Alves de Matos, Bruno Manadas, Leonor Saúde, Mariaelena D’Ambrosio, Pedro M. Costa

**Affiliations:** 1 Associate Laboratory i4HB, Institute for Health and Bioeconomy, NOVA School of Science and Technology, NOVA University of Lisbon, 2829-516 Caparica, Portugal; m.alcaide@campus.fct.unl.pt (M.A.); imf.cabral@campus.fct.unl.pt (I.M.C.); 2 UCIBIO—Applied Molecular Biosciences Unit, Department of Life Sciences, NOVA School of Science and Technology, NOVA University of Lisbon, 2829-516 Caparica, Portugal; 3 iMM—Instituto de Medicina Molecular, Faculdade de Medicina, Universidade de Lisboa, 1649-028 Lisboa, Portugal; lcarvalho@medicina.ulisboa.pt (L.C.); msaude@medicina.ulisboa.pt (L.S.); 4 CNC—Center for Neuroscience and Cell Biology, University of Coimbra, 3060-197 Cantanhede, Portugal; vera3m@gmail.com (V.M.M.); bmanadas@gmail.com (B.M.); 5 Centro de Investigação Interdisciplinar Egas Moniz (CiiEM), 2829-511 Caparica, Portugal; apamatos@gmail.com; 6 Instituto de Histologia e Biologia do Desenvolvimento, Faculdade de Medicina, Universidade de Lisboa, 1649-028 Lisboa, Portugal

**Keywords:** cnidaria, toxins, toxicity, marine bioprospecting, proteomics, histology, electron microscopy, bioinformatics

## Abstract

**Simple Summary:**

The unsurmountable diversity of marine life is an invaluable reservoir of natural compounds for drug discovery, amongst which toxins and other bioactive molecules in venoms and poisons are prized targets. Cnidarians are some of the best-studied marine venomous animals, yet most venom components remain unidentified. This study focuses on the sea anemone *Actinia equina* from Portuguese intertidal zones, comparing two common morphotypes, “green” and “red”, ultimately aiming to explore its potential as a source of bioactive compounds. We provide detailed examinations of *A. equina*’s anatomy and microanatomy, a proteomics analysis to identify proteinaceous toxins in its tentacles and toxicity testing on zebrafish embryos. The study confirms the presence of venom-injecting cells (nematocysts) in the tentacles but finds no differences between green and red varieties. Various toxins, including neurotoxins and pore-forming proteins, were discovered. Both green and red extracts exhibit toxicity to zebrafish embryos, with green anemones appearing more potent. Overall, this study unveils proteinaceous toxins in *A. equina* and demonstrates that different varieties harbour distinct bioactive compounds. Besides ecological considerations, these findings bring further promise to bioprospecting *A. equina* for novel toxins with potential biotechnological and biomedical interest.

**Abstract:**

The current study investigates the venom-delivery system of green and red morphotypes of the sea anemone *Actinia equina* to disclose its potential as a source of bioactive compounds. We compared the two morphotypes using electron and optical microscopy, proteomics, and toxicity assessment on zebrafish embryos. Specialized venom-injecting cells (nematocysts) are equally distributed and found in the tentacles of both varieties. Proteomics revealed proteins of interest in both red and green *Actinia*, yielding the three most abundant Gene Ontology (GO) terms related to the biological processes “proteolysis”, “hemolysis in another organism” and “lipid catabolic process”. Neurotoxins and cytolytic toxins similar to known cnidarian toxins like PsTX-60A and AvTX-60A, for instance, were identified in both types. Extracts from green and red anemones were toxic to zebrafish embryos, with green anemone venom appearing to be more potent. The findings highlight the presence of proteinaceous toxins in *A. equina* and the potential for different varieties to possess distinct bioactive compounds. Notably, pore-forming toxins are suggested for molecular probes and immunotoxins, making them valuable assets for potential biotechnological and biomedical purposes.

## 1. Introduction

Albeit little-explored compared to terrestrial ecosystems, the oceans’ immense biodiversity is an invaluable source of natural bioactives for drug discovery and biotechnology, with emphasis on toxins due to their potency and selectivity [1]. Animal poisons and venoms consist of a complex mixture of toxins (many of which are peptidic), salt and permeabilising enzymes. Toxins result from natural selection for disturbing vital systems of target organisms, such as their nervous and immune systems, blood coagulation and general metabolic homeostasis [2]. For instance, the first marine-derived product to be approved by the Food and Drug Administration (FDA) of the USA, “*Ziconotide*” (commercialized as *Prialt*), is a synthetic form of a ω-conotoxin from the poisonous cone snail *Conus magus*, used for treating severe and chronic pain [1,3,4]. More recently, “*Trabectedin*”, a DNA-binding agent derived from the marine tunicate *Ecteinascidia turbinata*, commercialized as “*Yondelis*”, has been approved in Europe for the treatment of soft-tissue sarcoma and ovarian cancer [5].

Cnidaria, a phylum of marine invertebrates that includes, for example, sea anemones, corals and jellyfish, known to be one of the oldest lineages of venomous animals [6], is of great interest in the search for new bioproducts and has provided knowledge of over 3000 marine natural products [7]. As soft-bodied animals, many of which are sessile, they rely on their venoms for protection from predators and the capture of prey [8]. The cnidarian venoms have not been completely described yet, but seem to contain various proteinaceous (peptides, enzymes and proteinase inhibitors) and non-proteinaceous compounds (purines, betaines and others) (see, for instance, the review by D’Ambra and Lauratano [9]). Although there are no cnidarian-derived drugs approved yet, some compounds are already being subjected to clinical trials. For example, ShK, a potassium-channel toxin isolated from the venom of the sea anemone *Stichodactyla helianthus*, was the source for “*Dalazatide*” (previously known as “ShK-186”), a synthetic peptide that is a specific inhibitor of the voltage-gated Kv1.3 potassium channel and has been subjected to trials for the treatment and management of autoimmune diseases such as psoriasis [10], rheumatoid arthritis [11], obesity and insulin resistance [12]. Most of the identified toxins, mainly classified as sodium- and potassium-channel-binding neurotoxins and pore-forming cytolysins, are found in specialized poisonous cells called cnidocytes. These cells are mostly located in the tentacles, but can also be found in the acrorhagi, aggressive organs lodged around the base of the tentacles acting as a defence mechanism for non-clonal anemones, and acontia, thin threads attached to the mouth or the body wall of the anemone for defence or the capture of prey [13]. The cnidae (or cnidocysts), membrane-enclosed cellular organelles belonging to the cnidocytes are the defining subcellular specialisation of the Phylum Cnidaria [6].

The object of this study is the sea anemone *Actinia equina*, a benthic cnidarian very common on the Portuguese rocky shores. Belonging to the Anthozoa class, these anemones have no medusa stage in their life cycle (the planktonic free-living stage); existing only as polyps (the benthic stage), *Actinia equina* is formed by a smooth column, typically red, green or brown, with a blue line on the edge of the base. The tentacles are located on the oral disc, surrounding the mouth of the polyp. This species is found in depths of about 20 m and the intertidal zones, attached to rocks or substrates by the basal disc, making them resistant to high temperatures and desiccation [14].

In *Actinia equina*, the cnidae, also referred to as nematocysts, manifest as either spined or un-spined threads devoid of hollow tubules or folds along their length [15]. Within the tentacles of *Actinia*, where they are most concentrated, nematocysts exhibit spines throughout the entire tube, serving primarily for prey capture and envenomation, as well as defence against predators through venom injection [16]. Conversely, nematocysts found in the acrorhagi lack spines and are employed in territorial defence, particularly against non-clonal conspecific anemones [17].

Previously, studies aimed to elucidate the composition of the venom of *A. equina*. The identified components, mostly proteinaceous, were classified as neurotoxins, cytolysins, and Kunitz-type peptides, responsible for the paralysis, immobilization, and death of the prey [18]. Also, the possibility of different colour morphs, corresponding to different species of sea anemones, has also been studied. In a study in the Isle of Man, the green colour morph from *A. equina* was considered a different species, *Actinia prasina* [19]. However, a similar study with animals from the Portuguese coast did not reveal sufficient genetic differences for the different colour morphs to be considered different species [20]. Despite this, there are no studies regarding the possible variability of venom composition between different varieties of *Actinia*. Although this species is not harmful to humans, the identification of these components and their mechanisms of action can be beneficial to developing effective treatments for cnidarian envenomation, as well as for developing new marine-derived drugs, biopesticides and molecular probes.

This work intends primarily to contribute to the study of the venom system of the sea anemone *A. equina* as a potential source of bioactive compounds with biotechnological potential for drug discovery. Specifically, this work aims to characterise the morphoanatomy of the venom-delivery apparatus; identify the main proteinaceous toxins and associated potential bioactives present in the venom; evaluate venom bioreactivity–toxicity; and compare the venom system between the two most common morphotypes (varieties) of *Actinia equina*, “red” and “green”.

## 2. Materials and Methods

### 2.1. Animal Collection

Live red and green *A. equina* were collected during low tide from the intertidal of the rocky shores in Cabo Raso, Cascais, Portugal (38°42′36.4″ N, 9°29′10.1″ W), in October 2021, and Costa da Caparica, Portugal (38°39′03.9″ N, 9°14′50.3″ W), in February and April 2022. The animals were transported alive in seawater to the laboratory and kept in a closed-circulation aquaria mesocosm fitted with constant aeration, water filtration and water recirculation. The water salinity, temperature and photoperiod were controlled at about 30, 20 °C, and a 16:8 h light–dark cycle, respectively. Animals were fed to the mouth twice a week with marine carnivore-pelleted food (Hikari). Animals were acclimatized for 7–14 days until processing.

### 2.2. Microscopy Analyses

Specimens were first anesthetized by submerging them in menthol-saturated artificial sea water and a few tentacles from red and green *A. equina* were fixed in 2.5% *v*/*v* glutaraldehyde (in 0.1 M cold phosphate-buffered saline (PBS), pH 7.4). Fixation was performed at room temperature for 2 h, followed by washing with PBS (3 × 15 min), overnight post-fixation in 1% *m*/*v* osmium tetroxide (OsO_4_) (in 0.1 M PBS, pH 7.4) and washing in Milli-Q-grade ultrapure water (3 × 10 min). The samples were dehydrated in a progressive series of acetone (30–100%), intermediately infiltrated with EPON resin–polypropylene oxide 1:2, 1:1 and 2:1 (30 min each) and finally embedded with EPON resin (Sigma-Aldrich, St. Louis, MO, USA). Semi-thin sections were obtained using a RM 2125 RTS rotatory microtome (Leica Microsystems, Wetzlar, Germany) equipped with a tungsten carbide blade. Histological analyses of these sections were performed using Toluidine Blue (TB) and a tetrachrome (TC) technique combining Alcian Blue for acidic mucins and sugars, Periodic acid-Schiff’s (PAS) for sugars and Weigert’s Haematoxylin and Picric Acid for muscle fibres and cytoplasm, respectively [21]. Observations were performed using a DM 2500-model microscope (Leica Microsystems, Wetzlar, Germany).

For observation by transmission electron microscopy (TEM), thin sections produced with an Ultramicrotome (Reichert-Jung Ultracut E) were collected onto copper mesh grids and stained with 2% *m*/*v* aqueous Uranyl Acetate and Reynold’s Lead Citrate [22]. Observations were conducted using a JEOL 100-SX-model TEM (Peabody, MA, USA) operated at 80 keV.

The external structure of whole specimens was, also, analysed by scanning electron microscopy (SEM). After fixation with 2.5% *v*/*v* GA (in 0.1 M PBS, pH 7.4), followed by post-fixation in 1% *m*/*v* osmium tetroxide (OsO_4_) (in 0.1 M PBS, pH 7.4), samples were dehydrated in a progressive series of acetone (30–100%). Then, samples were infiltrated with tert-butanol (3 × 15 min) at 40 °C and left to freeze overnight at 4 °C. The tert-butanol was sublimated under vacuum until the samples were completely dry [23]. The samples were mounted onto an aluminium plate with a copper ribbon for carbon coating using a JEOL JEE-400 vacuum evaporator (Peabody, MA, USA). Analyses were performed in a JEOL JSM-5400 scanning microscope (Peabody, MA, USA) operated at 20 keV.

### 2.3. Characterisation of the Venom Proteome

#### 2.3.1. Venom Extraction

Red and green sea anemones (*n* = 3) were retrieved from the rearing system and tentacles and oral discs were collected. The viscous liquid released in this process was added to the samples, avoiding the carryover of debris. The tentacles were then excised and macerated with scissors and plastic pestles. After that, the samples were centrifuged (15 min, ~12,000× *g* at 4 °C). The supernatant was transferred onto a fresh microtube, and cold Milli-Q-grade ultrapure water was added to the pellet to repeat the process of homogenization and centrifugation. The supernatants were centrifuged again (15 min, ~12,000× *g* at 4 °C) and the protein concentration was determined using a NanoDrop 2000 spectrophotometer (Thermo Fisher Scientific, Waltham, MA, USA). Protein precipitation was achieved by adding acetone to the sample to a final concentration of 80%, followed by storage at −20 °C until further use. The sample with the precipitate was then centrifuged (5 min, ~3000× *g* at 4 °C) and the acetone supernatant was removed. Finally, samples underwent lyophilization in a speed-vac for 20 min to eliminate traces of acetone (adapted from Macek and Lebez [24]). The protein extract was resuspended in a Tris-SDS buffer (0.5 M Tris-HCl, pH 7.0; 10% SDS).

#### 2.3.2. SDS-PAGE Analysis

The proteinaceous nature and quality of the extracts were assessed in a discontinuous gel system for the one-dimensional separation of the extracted proteins under denaturing conditions by sodium dodecyl sulphate-polyacrylamide gel electrophoresis (SDS-PAGE) [25]. Resolving and stacking gels contained 12% (*v*/*v*) and 6% (*v*/*v*) acrylamide, respectively. The running buffer consisted of 25 mM Tris, 192 mM glycine and 3.5 mM SDS, and the molecular standard used was NZY Colour Protein Marker I (Nzytech, Lisbon, Portugal), with a range of 5–245 KDa. The gels were stained with Coomassie Blue overnight and transferred to a destaining solution the following day.

#### 2.3.3. Toxin Identification by LC-MS/MS

The protein extracts were pooled into two samples, red and green, for the generation of an ion library, while 50 μg of protein from six samples (three red and three green) was used for the relative protein quantification. For all the samples, green fluorescent protein and maltose/maltodextrin-binding periplasmic protein were used as an internal standard solution. They ran in a Short GelC approach for LC-MS/MS analyses. The gel was then stained with Coomassie Blue. Each lane of the gel was cut into five fractions (in the case of the pooled samples) and into three (in the remaining samples) and left overnight for destaining and porcine trypsin digestion. Then, the peptides were analysed on a NanoLC 425 system (Eksigent, Dublin, CA, USA) coupled to a Triple TOF^TM^ 6600 mass spectrometer (Sciex, Framingham, MA, USA). The chromatographic separation through micro-LC was achieved in a Triart C18 Capillary Column 1/32” (12 nm, S-3 μm, 150 × 0.3 mm, YMC) at 50 °C. The flow rate was set to 5 μL/min, mobile phase A was 0.1% (*v*/*v*) formic acid plus 5% (*v*/*v*) DMSO in water and mobile phase B was 0.1% (*v*/*v*) formic acid plus 5% (*v*/*v*) DMSO in acetonitrile. The ionization source (ESI DuoSpray^TM^ from Sciex) was operated in the positive mode set to an ion spray voltage of 5500 V, 25 psi for nebulizer gas 1 (GS1) and the curtain gas (CUR). The rolling collision was used with a collision energy spread of 5. ProteinPilot 5.0.1 (Sciex) was used to execute peptide mass fingerprinting considering the following parameters: cysteine alkylation via acrylamide, digestion via trypsin and gel-based ID as a special factor. A customized database consisting of secreted toxin and venom-related reviewed proteins selected from UniprotKB (release 2022_01), plus any similar proteins from Cnidaria published elsewhere, even if unreviewed, was used to annotate the resulting amino acid sequences through sequence homology using Blast [26]. The percentage of sequence coverage and the number of matching peptides per protein (95% confidence) established the accuracy of the identification. Protein relative quantification was performed in the six samples by employing the SWATH processing plug-in for PeakView 2.0.01 (Sciex), while using the data from the protein identification. While peptide relative quantification was calculated by the sum of up to 5 fragments/peptides if they had an FDR < 1% for a minimum of two replicates of a minimum of one of the specimens, protein relative quantification was calculated by the sum of up to 15 peptides/proteins. The relative quantification of the internal standards was used for normalization.

### 2.4. Toxicity Testing

Red and green sea anemones (*n* = 3) were retrieved from the rearing system into separate Erlenmeyer, each pre-prepared with 10 drops of filtered and autoclaved seawater. Crude mucous secretions were harvested by the mechanical stimulation of the specimens for a few minutes. The mucus was then transferred to a fresh microtube, where buffer (Tris-HCl, pH 7.0 with 10% *m*/*v* L-dithiothreitol (DTT) and 1% *v*/*v* protease inhibitor cocktail) was immediately added (1:1). The samples were centrifuged (5 min, ~3000× *g* at 4 °C), and the supernatant was transferred to a fresh microtube, discarding the pelleted debris. The mucous samples were first filtrated with a 0.22 μm cellulose acetate syringe filter (FilterLab, Barcelona, Spain). Based on previously optimised protocols [27], the samples were fractionated and concentrated using Amicon Ultra centrifugal filters (Merck KGaA, Darmstadt, Germany). Two fractions were produced, >100 kDa filters and 3–100 kDa filters.

The extracts were then resolved through SDS-PAGE, as described above. The gels were stained with silver nitrate for increased sensitivity due to a low quantity of protein in the extracts. In brief, the gel was sensitized in 0.02% sodium thiosulfate solution for 1 min, followed by washing with Milli-Q-grade ultrapure water (3 × 20 s). Then, the gel was incubated in 0.1% silver nitrate solution for 20 min and washed with Milli-Q-grade ultrapure water (3 × 20 s). The gel was transferred into a new tray for development with a 3% sodium carbonate solution in the dark. Developing was halted by washing the gel in Milli-Q-grade ultrapure water for 20 s followed by immersion in 5% *v*/*v* acetic acid. The gel was stored in 1% *v*/*v* acetic acid.

The extracts were pooled in two samples, one for each morphotype, red (VM) and green (VE). Each sample consisted of a pool of three biological replicates. The buffer was exchanged for PBS using 500-μL 3 kDa Amicon Ultra centrifugal filters. Protein was quantified using Bradford’s method [28], using bovine serum albumin (Sigma-Aldrich) as standard. Absorbance was read at 595 nm in a Multiskan SkyHigh UV/Vis microplate spectrophotometer (Thermo Scientific).

Approximately 3 h post-fertilization, zebrafish embryos were dechorionated and exposed to different dilutions of the two pooled extracts (VM and VE) for 72 h in glass plates at 28 °C. The exposure concentrations were 0 μg/mL (control); 0.2 μg/mL (Dose 1); 0.4 μg/mL (Dose 2); 0.6 μg/mL (Dose 3); and 0.8 μg/mL (Dose 4) of total protein prepared in 5 mL of embryo medium. Each test was performed with 10 embryos, in triplicate. Every 24 h until the end of the bioassays, the surviving embryos were counted and their developmental status was recorded. 

### 2.5. Statistical Analysis 

Statistical analysis was conducted with R 4.2.1 [29]. The level of significance (α) was established at 0.05 for all analyses. Following the invalidation of assumptions for parametric analyses, namely normality and homoscedasticity (through Shapiro–Wilk’s and Levene’s tests, respectively), non-parametric statistics were employed. These include Kruskal–Wallis’ H test followed by Dunn’s test for post hoc comparisons. Log-logistic response curves and EC_50_ estimates were estimated for the longest time of observation (72 h) using a previously developed R-based application (BioModeller 1.01.05, with the required packages ‘MASS’, ‘drc’ and ‘tkrplot’).

Gene Ontology (GO) analyses were performed on proteins annotated by contrasting against the customized toxin database mentioned above, using the package UniprotR. Through this, we retrieved the protein families corresponding to the Accession IDs of the identified proteins, along with the GO terms associated with the Biological Process. This was executed for the Accession IDs of proteins exclusive to green specimens, exclusive to red specimens and common to both. The relative quantification of proteins was analysed using a Student’s *t*-test to compare between red and green varieties after the verification of normality and homoscedasticity.

## 3. Results

### 3.1. Morphoanatomic Characterisation of A. equina Venom-Delivery Apparatus

**Figure 1:**
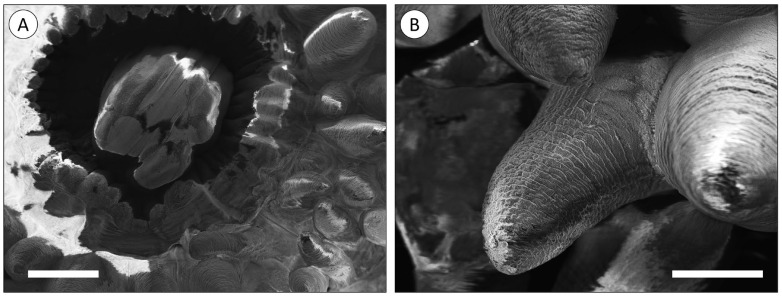
The external structure of *A. equina*. (**A**) SEM image of the mouth of the polyp surrounded by radially arranged tentacles. (**B**) SEM image of a tentacle. Scale bars: (**A**) 1000 nm, (**B**) 600 nm.

The mouth of the polyp is centred in the oral disc and the tentacles are arranged radially around the opening (Figure 1A). The tentacles were shown to be relatively short and conical despite artefactual shrinking, potentially caused by fixation (Figure 1B).

**Figure 2:**
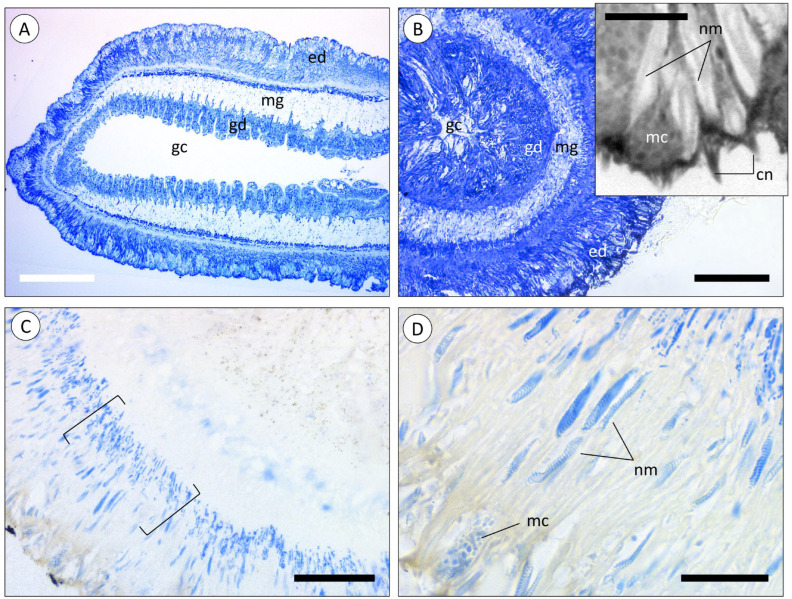
Microanatomy of the tentacles of *A. equina*. Longitudinal (**A**) and transversal (**B**) cross-sections of resin-embedded tentacles (Toluidine Blue stain). (ed) Epidermis, (gc) gastrovascular cavity, (gd) gastrodermis, (mg) mesoglea. Inset in (**B**): detail of the epidermis. (cn) Cnidocilium, (mc) mucocytes, (nm) nematocysts. (**C**) Transversal cross-section of a tentacle. Square brackets highlight the layer of cnidoblasts in the basal portion of the epidermis (Coomassie Blue stain). (**D**) Epidermis showing the presence of mucocytes (mc) and nematocytes (nm) (Coomassie Blue stain). Scale bars: (**A**) 200 µm, (**B**) 200 µm, inset 60 µm, (**C**) 50 µm, (**D**) 20 µm.

The tentacles of the sea anemone are extensions of the body, also presenting the gastrovascular cavity in the centre (Figure 2A). The inner cellular layer, directly in contact with the cavity, was identified as the gastrodermis (endodermal origin), responsible for the digestion and absorption of nutrients. The mesoglea, a cell-poor layer mostly comprised of connective tissue, separated the gastrodermis from the outer cellular layer, the epidermis (ectodermal origin), that lined the exterior of the polyp, tentacles included. The epidermis (Figure 2B, inset) was composed of epithelial cells intercalated with mucocytes and anchored in a layer of fibrils. The base of the cells appeared thinner, where the nuclei are located, whereas the extremity of the cells was enlarged and sac-like, packed with vesicles, and the margin of the cells were covered in microvilli. It was also in this extremity that cnidocytes seem to be observed, containing the nematocysts shown as an elongated body with a coiled-thread appearance. These were distributed throughout the whole epidermis layer and more densely packed in the outer extremity of the layer. These cells harbour cnidocilia, which are responsible for triggering the release of the nematocysts. 

The histochemical staining of the epidermis of a tentacle showed that nematocysts had a distinct composition from the surrounding tissue. The base of the epidermis cells was densely stained with Coomassie Blue, possibly indicating nematocyte precursor cells, the cnidoblasts (Figure 2C). The nematocysts, along with some vesicles present in mucocytes, were also stained with Coomassie Blue, indicating the presence of proteinaceous material (Figure 2D).

**Figure 3:**
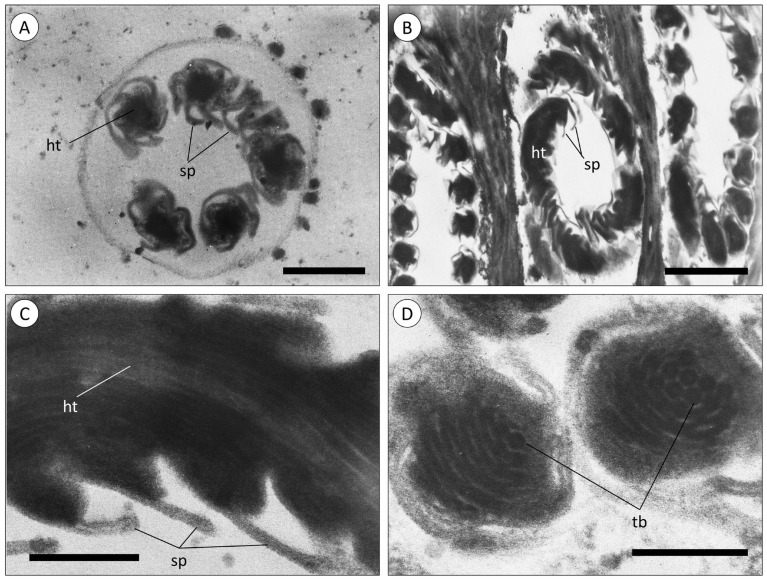
Transmission electron microscopy (TEM) of cnidae in the epidermis layer of tentacles of *A. equina*. (**A**, **B**) TNematocysts in transversal cross-sections of the tentacles of green specimens. (**C**) Nematocyst in a transversal cross-section of a red anemone. (**D**) A spirocyst in a transversal cross-section (red specimen). (ht) Hollow tubes, (sp) spines, (tb) hollow tubules. Scale bars: (**A**) 800 nm, (**B**) 1200 nm, (**C**) 240 nm, (**D**) 200 nm.

A closer examination of the cnidae present in the epidermis of tentacles in green and red specimens (Figure 3) confirmed the same structure of the nematocysts, consisting of a hollow tube with an even diameter and spines throughout. It was possible to identify another class of cnidae, the spirocysts (Figure 3D), characterised by the small tubules arranged helically, as described by Shick [17].

**Figure 4:**
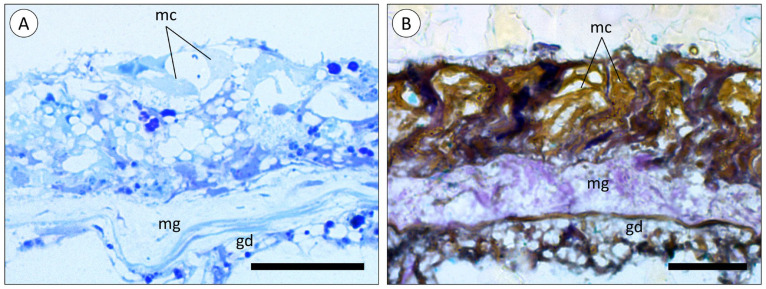
Microanatomy of the body wall of A. equina. (**A**) Transversal cross-section of a resin-embedded green specimen (Toluidine Blue). (**B**) Transversal cross-section of a resin-embedded red specimen (Tetrachromatic stain). (gd) Gastrodermis, (mc) mucocytes, (mg) mesoglea. Scale bars: (**A**), (**B**) 20 µm.

Similarly to tentacles, the body wall (“column” of the polyp) is composed of an epidermis and gastrodermis, separated by the mesoglea (Figure 4A,B). However, the epidermis of the body wall was not found to bare nematocysts, even though mucocytes were equally abundant.

### 3.2. Toxicity Testing

**Figure 5:**
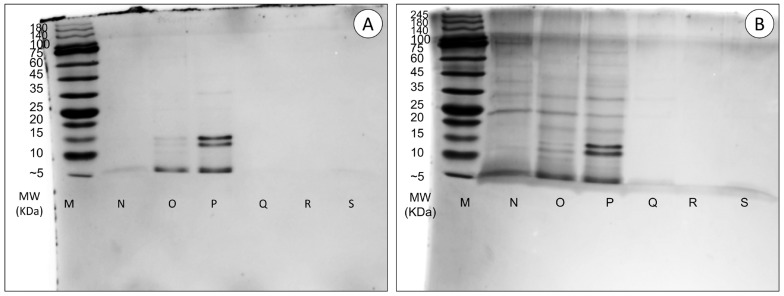
Separation by SDS-PAGE of protein extracts from *A. equina* stained with (**A**) Coomassie Blue and (**B**) Silver Nitrate. Each well was loaded with the same quantity of total protein (5.85 µg). Samples N, O and P belong to red specimens. Samples Q, R and S belong to green specimens. Sample M is the molecular marker.

Protein separation by SDS-PAGE (Figure 5) confirmed the proteinaceous nature of the extracts and showed major differences in band intensity between red and green specimens. Red specimen samples (N, O, and P samples in Figure 5) show some protein bands with a varying pattern that are not visible for green specimens. Overall, there were no significant signs of protein degradation, suggesting that the quantifiable peptidic material in these extracts corresponds to undisclosable proteins, potentially high-molecular-mass mucins that did not migrate beyond the stacking gel and may have contributed to block the migration of smaller molecular mass proteins.

**Figure 6:**
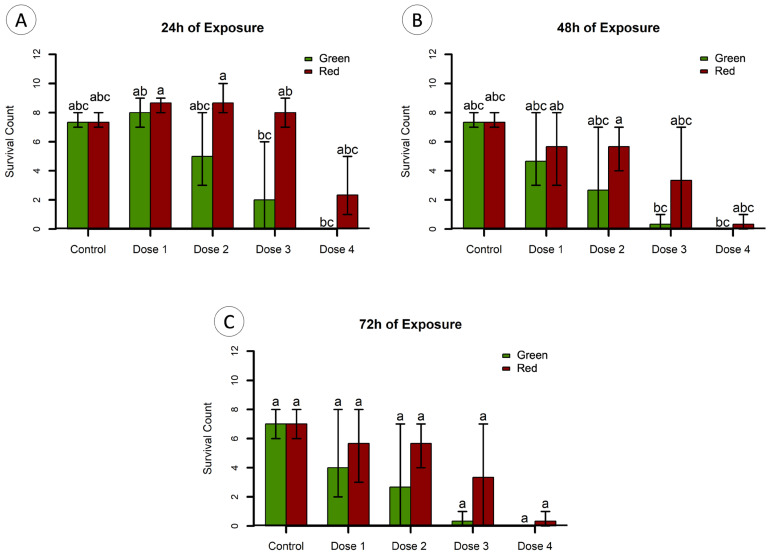
Zebrafish embryo toxicity testing of extracts from green and red *A. equina* morphotypes. The results are expressed as surviving individuals (as count) + standard deviation, from zebrafish embryos exposed to different doses of protein extracts, plus control (embryos exposed to the vehicle only, i.e., PBS) at (**A**) 24 h, (**B**) 48 h and (**C**) 72 h of exposure. Doses 1 (0.2 μg/mL), 2 (0.4 μg/mL), 3 (0.6 μg/mL) and 4 (0.8 μg/mL) refer to total protein in extracts per mL PBS. Different letters indicate significant differences between treatments (Dunn’s test *p* < 0.05).

The exposure of dechorionated zebrafish embryos to the pooled protein extracts showed that higher doses of protein (Doses 3 and 4) resulted in a significant decrease in the survival of zebrafish embryos at 24 and 48 h of exposure (Dunn’s test; *p* < 0.05). However, no significant differences were observed at 72 h of exposure (Dunn’s test; *p* > 0.05) (Figure 6C). At 24 h of exposure, significant differences were found for Dose 3 (0.6 μg/mL of total protein) of both extracts, but only the green extract in the higher dose, i.e., Dose 4 (Figure 6A). As for 48 h of exposure, significant differences were only found between lower doses (i.e., Doses 1 and 2) of the “red” extract and the higher doses (Doses 3 and 4) of the “green” extract (Figure 6B).

**Figure 7:**
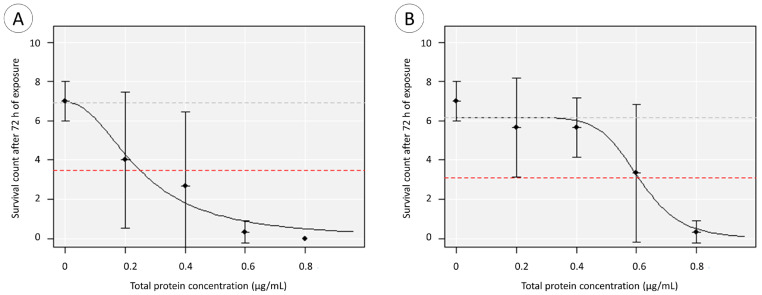
Acute toxicity of crude protein extracts from *A. equina* tentacles to zebrafish embryos (0–0.8 µg total protein per mL, in PBS). Survival was determined after 72 h of exposure to tentacle extracts from (**A**) green and (**B**) red morphotypes. The EC_50_ estimates are indicated by the dashed the red line. The results are given as mean ± SD from three independent experiments.

When testing the effect of morphotype on survival for each time of observation, significant differences between green and red extracts were only found at 24 h of exposure (*p* < 0.05). Moreover, when assessing for a dose–effect relationship at each time of observation, a similar result was obtained, as a significant correlation was only found for green extracts at 24 h of exposure, whereas for 48 h and 72 h of exposure, it was found for both extracts. An estimated EC_50_ of 0.25 (95% CI, 0.08–0.77) μg/mL was obtained for “green” (Figure 7A) and 0.61 (95% CI, 0.30–0.82) μg/mL for “red” extracts (Figure 7B).

The embryos still alive at 72 h of exposure showed signs of tail torsion, end-tail malformation, pericardial oedema and yolk sac oedema. These changes tended to occur more severely in embryos exposed to extracts from red specimens that exerted an inferior mortality rate when compared to extracts from green specimens.

### 3.3. Characterisation of Venom Proteins 

Proteomics on protein extracts yielded a total of 286 identified proteins (see Supplementary Materials). Of these, 68 were exclusive to red specimens, 102 to green specimens and 116 were common to both morphotypes (SI Tables S1–S3, respectively). A total of 44, 55 and 56 were identified as toxins or toxin-like proteins, respectively. Examples of these are presented in Table 1, Table 2 and Table 3. It is important to note that some of the toxins were specifically matched to *A. equina* proteins already in databases such as Delta-actitoxin-Aeq2a (which were common to both green and red morphotypes); Delta-actitoxin-Aeq2b, exclusive to green, and Delta-actitoxin-Aeq2b 2, exclusive to red. Most of the remaining toxins were identified in other cnidarians, such as *Actinia tenebrosa*, *Anemonia viridis* and *Anemonia sulcata* (Table 1). 

Despite the elevated number of hits against cnidarian toxins, some proteins were best-matched to the venom components of distant Eumetazoa, such as the Potassium channel toxin alpha-KTx 6.2, a 34 kDa, neurotoxin from the golden scorpion *Scorpio palmatus* (Table 2).

**Figure 8:**
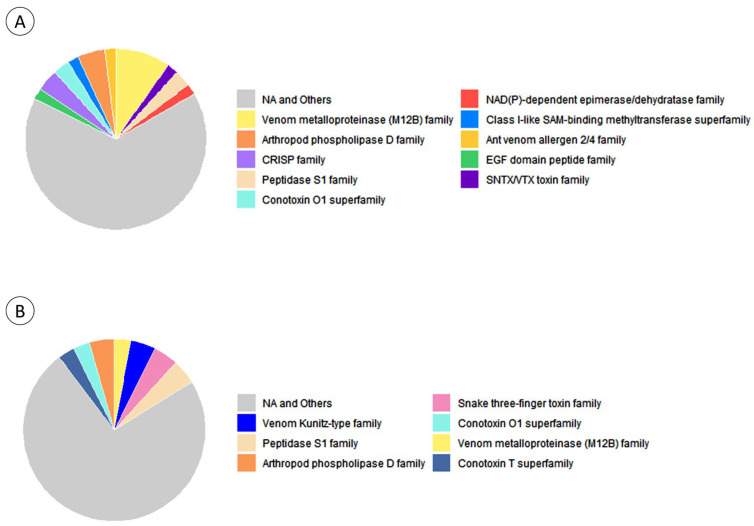
Most abundant toxins and venom-related protein families identified by homology matching in *Actina equina*. The results are segregated by protein extracts from (**A**) green and (**B**) red morphotypes.

Despite the proteins identified exclusively in green or red morphotypes, the categories “Venom metalloproteinase (M12B)”, “Peptidase S1”, “Conotoxin O1” and “Arthropod phospholipase D” were common to both groups (Figure 8). Proteins categorized as metalloproteinases were the most frequent in green-specimen extracts, whereas the “venom Kunitz-Type family” was dominant in protein extracts form red specimens. Generally, proteins exclusive to green or red morphotypes were categorized as toxins, enzymes or pathway-inducing peptides.

Statistical analysis on protein abundances, which exhibited important interindividual variation, yielded significant difference in the relative quantities of two proteins between red and green specimens (Student’s *t*-test; *p* < 0.05). These proteins were identified as similar to U-scoloptoxin (08)-Er5b (~11 kDa) and Deoxypodophyllotoxin synthase (>50 kDa), from centipede and plant origins, respectively, upregulated in green and red morphotypes, respectively. 

**Figure 9:**
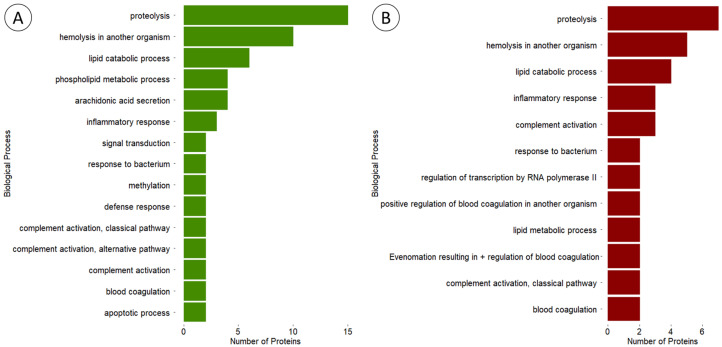
Comparison between the number of matched toxins and venom-related proteins from *Actina equina* associated with the most frequent ‘Biological Process’ category Gene Ontology (GO) terms. Results are shown for proteins exclusively identified by homology matching in extracts from (**A**) green and (**B**) red morphotypes.

The three most frequent GO terms within the “Biological Process” category for proteins exclusive to either morphotype were “proteolysis”, “haemolysis in another organism” and “lipid catabolic process” (Figure 9), and although bearing differences, most terms across both groups refer to biological processes capable of inducing or potentiating toxicity. However, different proteins associated with the same GO term may not have the same effect, since it only indicates the process in which the proteins are involved and not the specific mode of action of each one.

## 4. Discussion

The present study showed that *Actinia equina* possesses a distinct venom system situated within its tentacles, as evidenced by the absence of venom-delivery structures in the body wall of the animals. The existence of short and stout tentacles in *A, equina* implies that this species adopts an opportunistic suspension feeding strategy, preying on macrofauna. This involves feeding on organisms or detritus that descends onto their oral disc, rather than actively seeking out prey [30]. Also, *A. equina* is armed with two cooperating types of cnidae in this action: spirocysts and nematocysts. The spirocysts, lacking spines, contain small helically arranged tubules that create an adhering surface upon release, gripping the prey without penetrating. These have been described to be the numerically dominant cnidae when observed [17], suggesting the importance of spirocysts in the survival of cnidarians. However, the nematocysts, composed of a hollow tube bearing outward-directed spines, are the cnidae responsible for envenomation by injecting venom into the prey, as described for other cnidarians [15]. These findings suggest that the adhesion capabilities of spirocysts and the discharge of nematocysts work together to capture and immobilize prey. This cooperation might also be extended to mucus secretion from mucocytes, found surrounding the cnidae in the tentacles, as studies have found that mucus secreted by *A. equina* presents viscosity and osmolarity corresponding with hydrated adhesives with viscoelastic properties [31], potentiating the predation skills mentioned above. Note that mucocytes were also found lining the body wall of *A. equina*. However, along with the lack of cnidae in the body of the sea anemones, the mucocytes are morphoanatomically different from mucocytes found in the tentacles, which suggests that mucus secretion in the body wall serves a purpose apart from predation. The venom-delivery system showed no variation between green and red specimens, but such resemblance is seemingly not extended to potency and composition, with the two subjects likely being interlinked.

The toxicity assays suggest that *A. equina* secrete toxins that can exert negative effects on the prey with differing efficacy depending on morphotype. Venom extracts of *Actinia* have a toxicity effect on the survival of zebrafish embryos. Despite the low concentration, green specimen extracts exerted a faster toxic effect, resulting in a lower EC_50_, and, although rendering a lower mortality rate, red specimen extracts caused severe malformations to the surviving embryos, such as tail torsion and malformation, pericardial and yolk sac oedema. The complexity of venoms, which are mixtures of different compounds of which toxins are just a part, plus the variety of toxicological models render comparisons difficult. Nonetheless, a similar study with the zebrafish embryo model to test the toxicity of crude venom extracted from the snake *Montivipera bornmuelleri*, known as the Lebanon viper, yielded an LD_50_ (24 h) of 62 μg/mL of total protein in the venom extract [32], which means that *A. equina* venoms may be more toxic to the model, given the distinction in the toxicity threshold by several orders of magnitude (0.25 and 0.61 for extracts from green and red morphotypes, respectively), despite the differential duration of the assays. In addition, although the toxicity of the viper venom was hitherto tested in chorionated embryos (as opposed to the dechorionated embryos used in the present study), the referred study gives the understanding that the extracts obtained from *A. equina* are substantially toxic. Ultimately, the extracts tended to cause a similar effect as the assay progressed (i.e., leading to the full mortality of embryo batches), but the initial discrepancy is indicative of distinct venom potency and, therefore, its composition between green and red specimens of *A. equina*.

Among all marine invertebrates, cnidarians are one of the taxonomic groups for which a wide variety of proteinaceous toxins, and other venom bioactives have already been characterised and even proposed for potential biotechnological applications, despite the lack of consumer end products (refer to the recent reviews by Bordon et al. [33] and Amreen Nisa et al. [34]). The findings summarised in Figure 9 highlight some of the biological processes that may be modulated by components of the anemone’s venom, from haemolysis and inflammation to apoptosis and response to bacteria (see also the full list of matched proteins and respective accessions in Supplementary Materials). Altogether, the characterisation of the venom proteome revealed important clues to explain the distinct potency of the extracts from the two varieties, even if, at the present stage, further studies on protein characterisation and specific modes of action are needed. The identification rendered interesting proteins with biotechnological potential, with toxins being the majority. Delta-actitoxin-Aeq2a, a neurotoxin first characterized in *A. equina*, was identified in both green- and red-specimen extracts of the current study. This protein, also called Ae I, is a type-I sodium-channel inhibitory toxin that binds specifically to voltage-gated sodium channels, delaying their inactivation during signal transduction, and has been shown to have crab and mice toxicity [35]. Similar toxins, with at least 50% similarity, were found exclusively in red- or green-specimen extracts, such as Delta-actitoxin-Aeq2b 2 and Delta-actitoxin-Aeq2b, respectively. Even though further research on the isolated toxins is necessary, it implies that variance between *A. equina* varieties does exist. Additionally, the GO-term analysis revealed that both extracts have the same top three terms from the “Biological Process” search, but they are more frequent in the green-specimen extract, which is congruent with the heightened toxicity from this extract. Many of these terms referred to processes involved with toxicity; however, different proteins associated with the same process may not have the same mode of action, meaning that the actual role of the toxins in the observed toxicity would require further studies with the isolated toxins.

It is noteworthy that the analyses of *A. equina* proteins, according to SDS-PAGE (despite issues when resolving proteins from the green morphotype, likely due to mucins) and proteomic analyses, effectively confirm that this anemone secretes proteinaceous toxins and accompanying bioactives similar to other Cnidarian species. Albeit expected, this not only sustains the toxicity and conserved status of toxins of these animals, but also offers an important validation of proteomics and its findings. For instance, a protein similar to AvTX-60A, a 498-amino acid, 55.5 kDa, a toxin from the sea anemone *Actineria villosa*, was found exclusively in the red morphotype [36], whereas a protein similar to PsTX-60A, a 60 kDa cytolytic toxin isolated from the nematocysts of *Phyllodiscus semoni*, was exclusively identified in green anemones. The latter is believed to be one of the major causes of toxicity following stinging by *P. semoni*, resulting in dermatitis, local fever and ulceration in humans [37]. It must be highlighted that AvTX-60A and PsTX-60A are considered to belong to the same family containing membrane-attack complexperforin domains (MACPF), a common component of some potent cnidarian pore-forming proteins [36]. Nonetheless, the existence of this domain in pore-forming proteins from *A. equina* remains to be ascertained. As such, this example stands as illustrative of differences between morphotypes. In fact, pore-forming toxins (PFTs) are already relatively well-studied in *A. equina* and commonly referred to as actinoporins. Among these, a potent 20 KDa protein (LD_50_ to intraperitoneally injected in mice is described as 33.3 μg/kg) named Equinatoxin has already been isolated from tentacles of *Actinia* [38]. These PFTs have been proposed as molecular probes, as well as immunotoxins, to identify and damage certain cancer cells containing different profiles of sphingomyelin in the membrane, since actinoporins have a high affinity to this sphingolipid [39].

It must be noted that the extracts produced in the current study consisted of a mixture of proteins and not purified toxins, which by itself can imply that the quantities of each protein may fluctuate between extracts. Moreover, the extraction methods for toxicity assays and the molecular characterisation of the venom were different, and the resulting extracts were also distinct. Ultimately, the venom of *A. equina* is injected into the prey and not diffused, meaning that even though toxicity was tested on dechorionated zebrafish, the effects observed by exposure in the medium may be underestimated when compared to the natural venom injection by *Actinia equina*.

## 5. Conclusions

Marine bioactives present an important contribution to drug discovery, especially proteins, as these can provide safer, cost-effect and easily transformed compounds for biotechnological application and industrial scaled-up production. The characterisation of these marine products is crucial for the development of templates, allowing the synthetic reproduction of bioactivity while keeping the marine habitats untouched. This work showed that cnidarians, even though relatively unexplored, can be an important source not only to provide a vast selection of bioactive elements but also specific toxins capable of interfering with biological processes involved in vital systems of the organism, thus representing the potential of the phylum for drug discovery.

This study revealed that *Actinia equina* possesses a well-differentiated venom system, implying the secretion of toxins directed to specific targets. Indeed, several proteins were identified, such as neurotoxins, that can affect specific systems of the prey. Additionally, the extracted proteins caused a mortality of zebrafish embryos, validating the toxic nature of the venom of *A. equina*. One of the main findings of this work was the difference between red and green specimens, defined by the distinct toxicity efficacy of the extracts and the variance of protein identification between varieties. Furthermore, characterising the venoms allowed us to understand the interference of the proteins in biological processes targeted for toxicity, enhancing the species’ potential for the bioprospecting of cnidarian bioactives.

## Figures and Tables

**Table 1:** Examples of homology-matched toxins identified in extracts from both red and green morphotypes. Data include the respective species, the number of matched peptides (95% confidence) and the percentage of sequence coverage.

Protein Name	Accession	Species	Number of Peptides		% Coverage	
			Red	Green	Red	Green
Delta-actitoxin-Aeq2a	Q9NJQ2	*Actinia equina*	9	11	63.41	65.85
PI-actitoxin-Aeq3c	P0DMJ2	*Actinia equina*	8	6	87.72	87.72
PI-actitoxin-Aeq3a	P0DMW6	*Actinia equina*	5	5	77.97	77.97
U-actitoxin-Avd3n-like	A0A6P8IZ51	*Actinia tenebrosa*	1	2	12.5	12.5
PI-actitoxin-Aeq3a-like	A0A6P8HBL9	*Actinia tenebrosa*	4	4	62.85	62.82
U-actitoxin-Bgr3d	G0W2I1	*Bunodosoma granuliferum*	1	1	11.39	11.39

**Table 2:** Examples of homology-matched toxins exclusively identified in the green morphotype. Data include the respective species, the number of matched peptides (95% confidence) and the percentage of sequence coverage.

Protein Name	Accession	Species	Number of Peptides	% Coverage
Delta-actitoxin-Aeq2b	B1NWU4	*Actinia equina*	3	47.56
Delta-alicitoxin-Pse2a	P58911	*Phyllodiscus semoni*	1	1.80
Delta-alicitoxin-Pse2b	P58912	*Phyllodiscus semoni*	1	3.07
U-actitoxin-Avd9c	P0DN02	*Anemonia viridis*	1	21.25
Potassium channel toxin alpha-KTx 6.2	P80719	*Scorpius palmatus*	1	35.29
U16-lycotoxin-Ls1a	B6DD52	*Lycosa singoriensis*	1	8.54

**Table 3:** Examples of homology-matched toxins exclusively identified in the red morphotype extract. Data include the respective species, the number of matched peptides (95% confidence) and the percentage of sequence coverage.

Protein Name	Accession	Species	Number of Peptides	% Coverage
U-actitoxin-Avd3s (Fragment)	P0DN20	*Anemonia viridis*	4	41.25
Delta-actitoxin-Aeq2b 2	B1NWU3	*Actinia equina*	3	45.12
KappaPI-actitoxin-Ael3a	P86862	*Anthopleura elegantissima*	2	13.85
Type III potassium channel toxin protein	A0A0S1M193	*Anemonia sulcata*	2	18.67
Delta-thalatoxin-Avl2a	Q76DT2	*Actineria villosa*	2	5.42
Phospholipase A2 A2-actitoxin-Ucs2a	A7LCJ2	*Urticina crassicornis*	1	6.45

## Data Availability

Data are provided in the manuscript and annexed in the Supplementary Information. Further information can be provided per request.

## Supplementary Materials

The following supporting information can be downloaded at: https://www.mdpi.com/article/10.3390/ani14060981/s1. It contains the list of annotated proteins through homology matching using BlastP. The file contains a list for each morphotype and a list from proteins in common in s separate spreadsheets.
